# Isolation Enhancement of a Two-Monopole MIMO Antenna Array with Various Parasitic Elements for Sub-6 GHz Applications

**DOI:** 10.3390/mi13122123

**Published:** 2022-11-30

**Authors:** Yitao Liu, Zhuo Yang, Ping Chen, Jun Xiao, Qiubo Ye

**Affiliations:** School of Ocean Information Engineering, Jimei University, Xiamen 361000, China

**Keywords:** multiple-input/multiple-output (MIMO) antenna, mutual coupling, parasitic element, T-shaped ground branch, isolated branch

## Abstract

In this paper, a high-isolation multiple-input/multiple-output (MIMO) microstrip monopole antenna array is investigated. To reduce the mutual coupling between antenna elements, a novel composite parasitic element constituted by a T-shaped ground branch and an isolated branch was designed and analyzed. The proposed composite parasitic element is capable of generating a unique three-dimensional weak electric field, which can effectively suppress the mutual coupling between the antenna elements. To give an intuitive illustration about the design principle and decoupling strategy of the proposed antenna, the antenna design procedure was ingeniously divided into four steps, and three types of decoupling structures during the antenna evolution were meticulously analyzed at both the theoretical and the physical level. To validate the proposed decoupling concept, the antenna prototype was fabricated, measured, and evaluated. The reflection coefficient, transmission coefficient, radiation pattern, and antenna gain were studied, and remarkable consistency between the measured and simulated results was observed. The simulations showed that the antenna has a peak gain of 3.5 dBi, a low envelope correlation coefficient (ECC < 0.001), and a high radiation efficiency (radiation efficiency > 0.9). Parameters of the proposed MIMO antenna including electrical dimension, highest isolation level, and 20 dB isolation bandwidth were evaluated. Compared with the previous similar designs, the proposed antenna exhibits attractive features including compressed dimension (0.55λ_0_ × 0.46λ_0_), extremely high isolation level (approximately 43 dB), fabulous 20 dB isolation bandwidth (3.11–3.78 GHz, 19.4%), a high diversity gain (DG > 9.99 dB), an appropriate mean effective gain (−3.5 dB < MEG < −3 dB), and low design complexity.

## 1. Introduction

With the rapid development of the wireless communication industry, the world is coming into the fifth generation (5G) era. In 2020, 5G wireless system began commercial use [1]. In this background, unprecedented data rate, capacity, and latency have been demanded in wireless communication. Owing to the merits of the higher data rate, larger capacity, lower latency, and immunity to multipath fading, the multiple-input/multiple-output (MIMO) antenna has been an indispensable part in 5G communication systems [2]. However, the 5G terminals or platforms with tight space restriction lead to a gradual narrowing of the distance between antenna elements. According to the antenna array theory, the mutual coupling among antenna elements cannot be negligible when the arrangement distance of antenna elements is less than a half wavelength, which can deteriorate antenna performance, including impedance bandwidth, radiation pattern, and efficiency [3,4,5]. Consequently, the mutual coupling among antenna elements has been an inevitable issue in compact MIMO antenna design, especially in application with constrained space.

To mitigate the mutual coupling between antenna elements, several effective decoupling techniques have been proposed in the past decades, which can be roughly classified into three categories according to the decoupling mechanism [6]. The first type is destructing the coupling path by suppressing the coupling current or wave between antenna elements directly. As a typical representative, the defected ground structure (DGS) [7,8,9,10], possessing a band-stop response, can effectively block the propagation of surface waves by perturbing the current distribution on the antenna ground, but the DGS usually suffers from the drawback of high front-to-back ratio (FBR). Fortunately, the development of various metastructures provides new insight into suppressing the coupling current or wave between antenna elements due to their unique electromagnetic property at certain frequency range, such as single negative metamaterial (SNG) [11,12], antenna decoupling surface (ADS) [13,14], frequency selective surface (FSS) [15,16], polarization conversion surface (PCS) [17], metasurface structure [18,19], and electromagnetic bandgap (EBG) structure [20,21,22,23]. In [15], the proposed metasurface structure was inserted between MIMO antenna elements to reduce the coupling. This metasurface structure is regarded as a spatial band-stop filter to suppress the spatial electromagnetic wave of the elements. However, the metasurface isolation wall has the disadvantage of narrow bandwidth with high price. In [17], a novel antenna decoupling surface was designed to tackle the mutual coupling issue in large-scale antenna array systems by generating out-of-phase reflected waves to counteract coupling waves. However, the antenna decoupling surfaces were deposited approximately a half wavelength above the antenna surface, which increased the antenna profile tremendously. In [19], a 1 × 2 polarization microbase-station antenna based on a metasurface isolation wall was designed. The novel isolation wall composed of dual metasurfaces with the ground was used to reduce coupling between antenna arrays of microbase-stations. However, the cost of the metasurface structure is much higher than that of the ordinary copper patch for improving the same isolation. In [22], a novel fence-type decoupling structure was proposed, which was mainly composed of several slits with equal size. The multiple slits acted as a band-stop filter, effectively reducing the coupling current between two ports. However, the introduced decoupling structure affects the impedance matching at low frequency, and the manufacturing process is complicated.

The working mechanism of the second type of decoupling approach depends on the cancellation of the original coupling path between antenna elements and the extra coupling path introduced by employing the neutralization line (NL) [24,25,26,27], the decoupling network (DN) [28,29,30], or the parasitic element (PE) [31,32,33,34]. Both NLs and DNs, consisting of microstrip circuits and lumped elements, are directly connected to antenna elements, which can provide the decoupling current with equal amplitude and opposite phase to counteract the coupling current. However, as the interconnector in antenna circuits, both NLs and DNs increase the difficulty of impedance match and introduce additional insertion loss, which will decrease antenna efficiency [30]. Compared to the above method, the parasitic element can provide an alternative way to create an indirect coupling path, which is placed in the vicinity of antenna elements without physical connection. When the antenna is excited, an induced current appears in the parasitic element with equal amplitude and opposite phase to counteract the coupling current. However, the field radiated by the induced current in parasitic element distorts the original radiation pattern of the antenna [33]. Until now, the most reported decoupling structures have typically need extra space between antenna elements or vertically above the antenna surface, which inevitably increases the antenna profile and system complexity. In addition, the applications of decoupling structures are always accompanied by the insertion effect, which degrades the comprehensive performance of antennas, including impedance match, radiation efficiency, or pattern.

Therefore, to overcome the above deficiencies of relying on decoupling structures, a third type of decoupling approach has been exploited, where the decoupling concept is that the MIMO antenna possesses a high natural isolation after design [35,36,37,38,39,40,41]. The typical representative decoupling approaches based on this concept include pattern diversity [35,36], polarization diversity [37,38], self-decoupling [39,40], and the theory of characteristic modes [41], which have a distinguished advantage that the antenna performance is almost unchanged since no additional structure is positioned in or around the radiating structures. However, the polarization or pattern diversity always has rigorous limitations on antenna structures, arrangement, and feeding methods. Despite the fact that the self-decoupling method is more flexible and ingenious than polarization or pattern diversity, this method lacks a systematic design guideline and versatility, attributed to the self-decoupling effect usually being realized by taking advantage of the inherent property of antennas. In [38], parasitic elements employing polarization diversity were used to reduce mutual coupling. However, the decoupling effect of the polarization diversity is not suitable for broadband antennas. In [40], a weak field-based self-decoupling technique was proposed. The working mechanism involves arranging the adjacent element in the area of the weak field generated by two fields excited by feeding structure and radiating patch which cancel each other. Nevertheless, this decoupling technique is only suitable for inset-fed patch antenna arrays.

In this paper, a two-element monopole array with high isolation is proposed and developed. According to a thorough study concerning the working mechanism of various parasitic elements, two types of parasitic elements including the T-shaped ground branch and the isolated branch are employed to suppress the mutual coupling between antenna elements. The T-shaped ground branch is located in the middle line of the antenna ground while the isolated branch is integrated into the substrate of antenna. The main novelties and merits of this study are as follows:

(1) Unlike past parasitic elements devoted to MIMO decoupling, the combination of the T-shaped ground branch and the isolated branch can be regarded as a three-dimensional composite parasitic element. The field generated by the induced current in the T-shaped ground branch can counteract the surface coupling field between antenna elements, while the isolated branch can eliminate the coupling field inside the substrate. Consequently, a unique three-dimensional weak field is created between two monopole elements, which has the distinguished ability to suppress the electromagnetic interference between antenna elements. Simulated results indicate that the highest isolation level of the final designed MIMO antenna exceeds 42.5 dB, MIMO isolation within the whole operating band is over 20 dB, and the average isolation improvement is approximately 16 dB compared to the original antenna.
(2) Since the T-shaped ground branch shares a common microstrip stem with the isolated branch integrated into the substrate of antenna, the proposed antenna has a comparatively compact structure, where only a single PCB is employed in antenna design. The overall size of the proposed antenna is 0.55λ_0_ × 0.46λ_0_ × 0.018λ_0_ (λ_0_ is the free space wavelength at the center frequency).

The remainder of this paper is organized as follows: Section 2 provides the design details of the MIMO antenna, including antenna configuration, design procedures, the decoupling concept, and theoretical analysis of the simulation results. The effects of some crucial parameters of decoupling structure on antenna performance are studied in Section 3. In Section 4, the measurement results of the fabricated antenna prototype are presented and discussed. A comprehensive comparison with previous similar studies and a discussion are given in Section 5. Lastly, a brief conclusion is drawn in Section 6.

## 2. MIMO Design

### 2.1. MIMO Antenna Configuration

**Figure 1:**
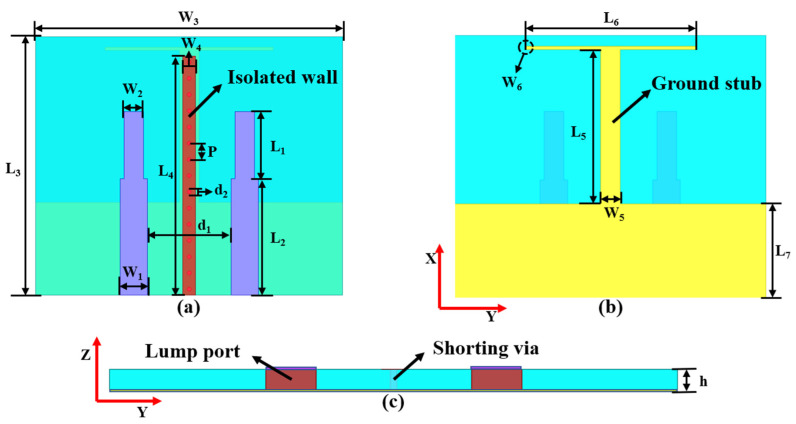
The configuration of the proposed MIMO antenna: (**a**) top view, (**b**) bottom view, and (**c**) side view.

The configuration of the proposed MIMO antenna is shown in Figure 1. It consists of two metal layers printed on the top and bottom of the FR4 substrate with relative permittivity of 4.4 and loss tangent tan δ = 0.02. The top metal layer is the physical structure of the designed monopole element, which is constituted by the feed line and radiating structure. The radiating structure is a simple open microstrip line which is directly fed by a wider microstrip line. Conspicuously, the width of the feed line is slightly larger than that of the radiating structure, which is for the sake of realizing fabulous impedance match. In addition, to obtain better radiation performance, the length of the feed line is slightly longer than the width of the ground. The two monopole elements are identical in terms of structure parameters which are symmetrically distributed on two sides of the top layer with respect to the *x*-axis. The ground of the MIMO antenna is located on the bottom layer, which is the evolution of the antenna element ground loaded with the T-shaped ground branch. The T-shaped ground branch consists of an I-shaped ground branch placed along the central line of the substrate and a thinner horizontal ground stub, which is symmetrically added to the end of the I-shaped ground branch along the *y*-axis. The isolated branch consists of three parts: a parasitic strip, shorting vias, and a microstrip stub shared with the T-shaped ground branch. A parasitic strip is printed on the back side of the I-shaped ground branch, which is connected to the I-shaped ground branch by utilizing a row of periodic shorting vias with center-to-center distance *p* and diameter of *d*_2_. Owing to the T-shaped ground branch and the isolated branch sharing a common microstrip stub, the combination of the T-shaped ground branch and isolated branch can be regarded as a composite three-dimensional parasitic element which is symmetrical with respect to the central line of the substrate. In this design, a full wave electromagnetic simulation software high-frequency structure simulator (HFSS) is used to analyze and optimize the proposed antenna. Two lumped ports with 50 Ω are utilized in the simulation. The final dimensions of the proposed antenna are listed in Table 1.

### 2.2. MIMO Antenna Design Procedures

**Figure 2:**
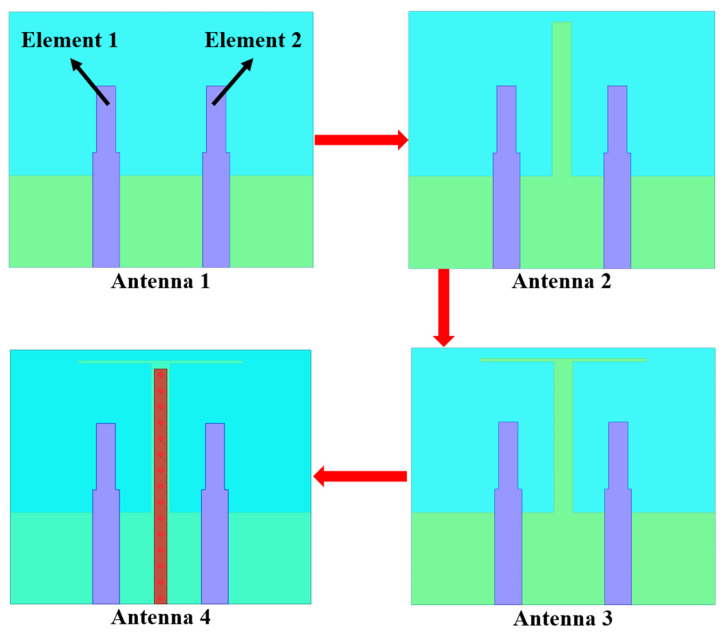
The design evolution of the proposed MIMO antenna.

To clarify the design principle and working mechanism of the proposed MIMO antenna, the antenna design evolution is elaborately depicted in Figure 2, which can be divided into four steps. For convenient comparison and analysis, the corresponding four antennas in antenna evolution process are expressed as antennas 1, 2, 3 and 4, respectively. Initially, a two-monopole element array is designed without any decoupling structure, which is marked as antenna 1. Secondly, an I-shaped ground branch is loaded at the center of the ground to reduce the mutual coupling between antenna elements and the newly generated antenna is named as antenna 2. After that, a thin horizontal stub is added to the terminal of the vertical stub to form a T-shaped ground branch in antenna 3. Lastly, periodic metallic shorting vias and a parasitic strip are employed to construct the isolated branch to eliminate the coupling field existing inside the substrate, and antenna 4 is constructed. Subsequently, the proposed high isolation MIMO antenna is accomplished. It is noteworthy to mention that all available sizes from antenna 1 to antenna 4 are consistent for convenient comparison.

### 2.3. Simulation Results of the MIMO Antennas

**Figure 3:**
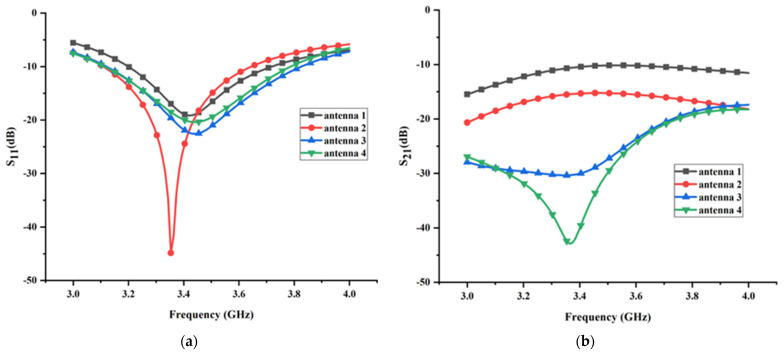
(**a**) The reflection coefficient of antenna 1–4; (**b**) the transmission coefficients of antenna 1–4.

According to the aforementioned discussion, the decoupling structure has experienced two significant adjustments. One is the one-dimensional ground stub transformed into a two-dimensional T-shaped ground branch, and the other is the T-shaped ground branch integrated with the isolated branch to construct a composite parasitic element with complicated three-dimensional structure. To illuminate the operation mechanism and decoupling effect of the above three types of decoupling structures, the simulation results in antenna design procedures, including the reflection and transmission coefficient, were analyzed meticulously step by step. The reflection and transmission coefficient linked to antennas 1–4 are shown in Figure 3a,b, respectively. It is worth mentioning that all simulation results are obtained with the port of element 1 excited while the port of element 2 is terminated by 50 Ω match load. Under this condition, the values of *K*_21_ and *K*_2*d*_ are equal to 0, which do not need to be considered in analysis such that the difficulty of analysis is immensely diminished.

### 2.4. Decoupling Concept

**Figure 4:**
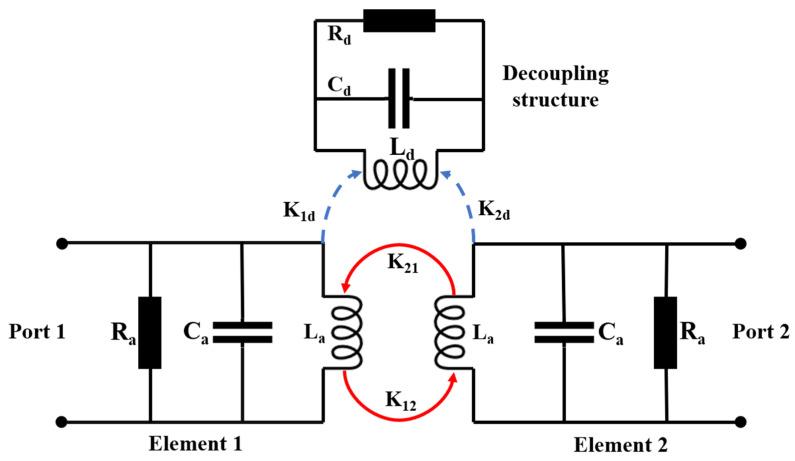
The equivalent circuit model of the proposed MIMO antenna.

As mentioned above, the decoupling concept of parasitic elements is the introduction of an additional coupling path to counteract the original coupling path existing between antenna elements, which can be explained on the basis of an equivalent circuit model. The equivalent circuit model of the proposed MIMO antenna is provided in Figure 4 for qualitative analysis, where both the antenna element and decoupling structure are equivalent to a parallel RLC resonator circuit. *R_a_* is the radiation resistance of the antenna element, while *R_d_* represents the ohmic loss of the decoupling structure. *L_a_* and *C_a_* are used to describe the equivalent inductance and capacitance of antenna element, respectively. Similarly, *L_d_* and *C_d_* are utilized to describe the equivalent inductance and capacitance related to the total decoupling structure. When the antenna elements are excited, the coupling wave may propagate between them, resulting in the electromagnetic energy coupling to the decoupling structure and adjacent antenna element.

Hence, electromagnetic energy can be used to quantitatively analyze the decoupling process [42]. According to the coupling mechanism of the electromagnetic energy, the coupling can be divided into two categories as capacitive coupling and inductive coupling. As mentioned above, the physical structures of antenna element and decoupling structure are constituted by the microstrip line and metallic shorting vias. Additionally, the distribution distance of antenna element and decoupling structure is large enough. Consequently, the coupling between antenna element and decoupling structure, and the mutual coupling between antenna elements are inductive coupling.

Therefore, there are two kinds of coupling in the MIMO antenna. One is the coupling between the antenna element and decoupling structure, while the other is the mutual coupling between antenna elements. Because the decoupling structure is the passive device, the transmission of the electromagnetic energy is mainly from the antenna elements to the decoupling structure. The type of coupling between antenna element and decoupling structure can be basically identified as unidirectional coupling. In Figure 4, *K*_1*d*_ and *K*_2*d*_ represent the coupling energy from the two monopole elements to the decoupling structure. On the other hand, the transmission direction of electromagnetic energy among two monopole elements is bidirectional. Consequently, *K*_12_ and *K*_21_ represent the coupling energy between two antenna elements.

Because of the symmetrical structure of the proposed MIMO antenna, the coupling mechanism of element 1 and element 2 is the same, it can be easily inferred that *K*_12_ is equal to *K*_21_ and *K*_1*d*_ is equal to *K*_2*d*_. Thus, we only analyze the decoupling principle when element 1 is excited. After loading the decoupling structure, the coupling energy which is exchanged between element 1 and other part is *K*_1*d*_ and *K*_12_. The cancellation of coupling energy can be expressed as

*K*_12_ + *K*_1*d*_ = 0. (1)

If *K*_1*d*_ and *K*_12_ are phase-reversed with similar amplitudes by loading appropriate decoupling structures, the two couplings can be almost canceled.

According to the circuit theory, the coupling between two inductors mainly depends on their size, structure, and separated distance. In the antenna design process, the antenna element structure and separated distance are fixed. Consequently, the value of *K*_12_ varies slightly. Instead, the value of *K*_1*d*_ is changed dramatically when the decoupling structure is adjusted significantly at each design step. Therefore, to eradicate the mutual coupling between antenna elements entirely, it is essential to design the structure of parasitic element deliberately to control the amplitude and phase of *K*_1*d*_ so as to satisfy Equation (1).

### 2.5. Investigation of the MIMO Antennas in Different Design Phases

#### 2.5.1. The Antenna without Decoupling Structure

**Figure 5:**
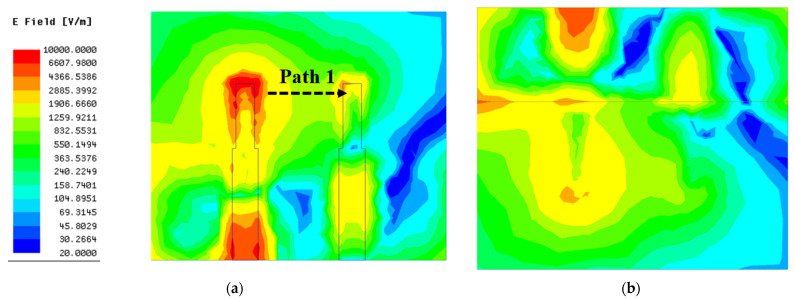
The surface electric field distribution of antenna 1: (**a**) top surface; (**b**) bottom surface.

As shown in Figure 3, the working frequency range (S_11_ < −10 dB) of the antenna without a decoupling structure is from 3.2 GHz to 3.7 GHz, with a relative impedance bandwidth of 15.4% and a minimum reflection coefficient of −19 dB. Correspondingly, the transmission coefficient of antenna without decoupling structure in the working frequency range is extremely close to −10 dB, indicating that strong mutual coupling exists between antenna elements. To visualize the mutual coupling between antenna elements, the surface electrical field distribution of the antenna without a decoupling structure at resonant frequency is presented in Figure 5. It can be seen clearly that an apparent coupling path (black dotted arrow in Figure 5) exists between two monopole elements. Hence, when element 1 is excited, the strong coupling electric field appears in the feed line, radiating structure, and ground of the element 2, as shown in Figure 5.

#### 2.5.2. The Decoupling Effect of the I-Shaped Ground Branch

Obviously, the value of *K*_1*d*_ is zero when the decoupling structure is absent in antenna design, resulting in Equation (1) not being satisfied. In order to suppress the mutual coupling between the antenna elements, it is necessary to introduce an additional coupling path to counteract the original coupling path existing in the antenna without decoupling structure. The I-shaped ground branch, a branch of the parasitic element, possesses the advantages of simple structure and design flexibility, which protrudes from the middle of the ground edge to introduce an extra coupling path between antenna elements and the I-shaped ground branch. Looking at the transmission coefficients of original antenna and antenna with the I-shaped ground branch, a slight reduction in transmission coefficient appears after loading the I-shaped ground branch, which demonstrates that employing the I-shaped ground branch to suppress the mutual coupling between the antenna elements is feasible.

**Figure 6:**
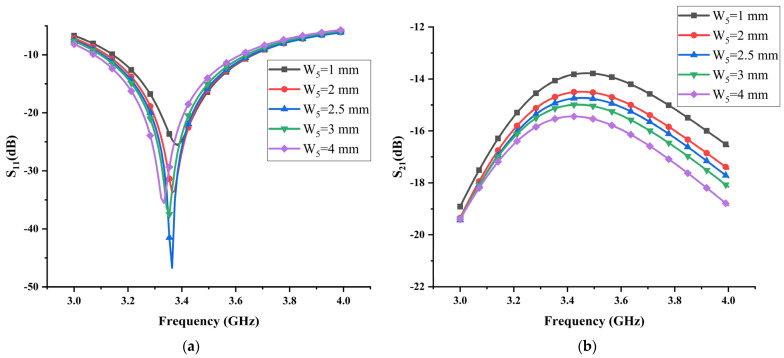
The effects of the width of the I-shaped ground branch $W_{5}$ on (**a**) reflection coefficient and (**b**) transmission coefficient.

The influence of different widths of the I-shaped ground branch is remarkable on reflection coefficient and transmission coefficient. It can be seen from Figure 6a that, while increasing the width of the I-shaped ground branch, the antenna’s working frequency is shifted to the left. Figure 6b shows that the isolation between antenna elements is gradually enhanced with the increase in the width of the I-shaped ground branch. When $W_{5}=4\;\mathrm{mm}$, the antenna deviates from the required working frequency. By contrast, the optimal isolation can be obtained only when the width of the I-shaped ground branch $W_{5}=3\;\mathrm{mm}$.

**Figure 7:**
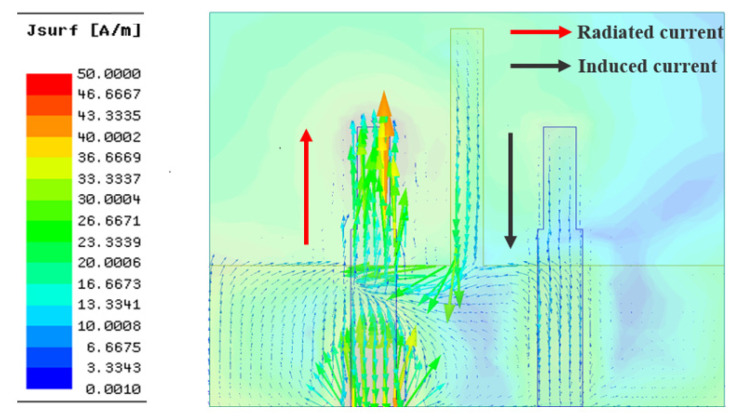
The distribution of surface current vectors of antenna 2.

**Figure 8:**
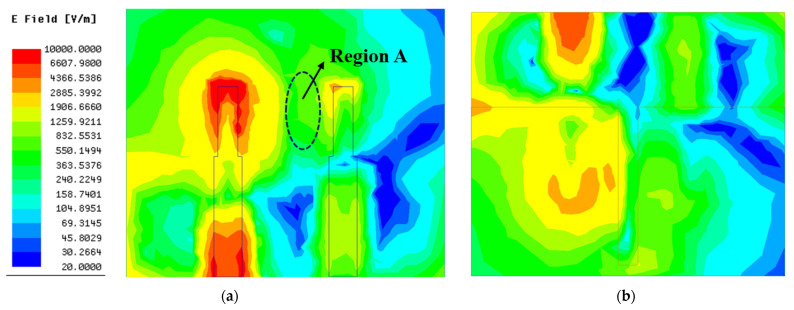
The distribution of the surface electric field of antenna 2: (**a**) top surface; (**b**) bottom surface.

To give an intuitive explanation for the working mechanism of the I-shaped ground branch at the physical level, the surface current vector and electric field distributions of antenna with the I-shaped ground branch are exhibited in Figure 7 and Figure 8, respectively. As shown in Figure 7, after loading the I-shaped ground branch, partial electromagnetic energy may couple to it, leading to a reverse induced current on the surface of the I-shaped ground branch. The induced current is along the direction of black arrow. It is believed that the field generated by the reverse induced current cancels out with partial coupling field derived from the current located on the radiation structure of the monopole element. The current on the radiation structure is along the direction of the red arrow. Hence, the amplitude of the coupling field in the region A (marked by the elliptical dotted line in Figure 8 is decreased slightly and the original coupling existing between antenna elements is weakened, immediately resulting in a slight decline in coupling field intensity on the surface of the radiation structure, feed line, and ground of element 2. Moreover, the I-shaped ground branch also affects the reflection coefficient of the original antenna. As shown in Figure 3a, the working frequency range (S_11_< −10 dB) of the antenna loading with the I-shaped ground branch is 3.11 GHz to 3.64 GHz, with a relative impedance bandwidth of 15.7% and a minimum reflection coefficient of −44.9 dB. Compared with antenna without the decoupling structure, the resonant frequency of the antenna loading with the I-shaped ground branch is shifted toward to the lower frequency, and the impedance matching is superior to the original antenna, which indicates that the I-shaped ground branch has a significant impact on antenna performance and effectively alleviates the negative effect of mutual coupling on antenna impedance matching.

#### 2.5.3. The Decoupling Effect of the T-Shaped Ground Branch

**Figure 9:**
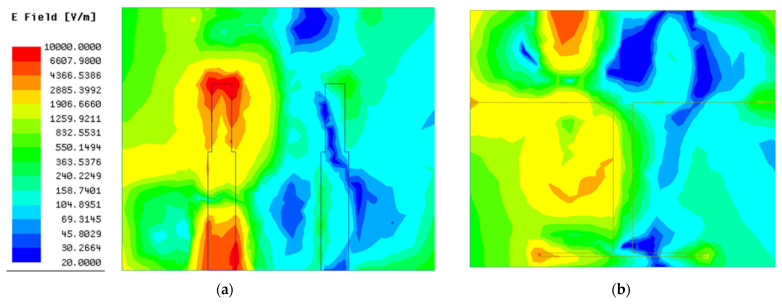
The distribution of surface electric field of antenna 3: (**a**) top surface; (**b**) bottom surface.

Despite the fact that the I-shaped ground branch can improve the isolation of the MIMO antenna, it is insufficient. With reference to the original antenna, only approximately 5 dB isolation improvement is attained after the antenna is loaded with the I-shaped ground branch, which is mainly caused by the following two factors: (1) the intensity of the induced current on the surface of the ground stub is too weak and, thus, the field generated by the induced current cannot counteract the coupling field between antenna elements entirely; (2) the induced current is mainly concentrated along the x direction and, thus, the field generated by the induced current cannot cancel out with the coupling field distributed along the y direction. Due to the above two factors, the intensity of the remaining coupling field between antenna elements is still too large. In other words, the amplitude of the coupling energy *K*_1*d*_ is too low compared with *K*_12_. Consequently, to further reduce the mutual coupling between antenna elements, two crucial requirements associated with the induced current located on the decoupling structure should be satisfied. One is that the intensity of the induced current should be in proximity to the coupling current. The other is that the distribution area of the induced current should be as large as possible to counteract the coupling current. To meet the above two requirements, a thin horizontal stub is added to the end of the ground stub to form a T-shaped ground branch in antenna 3, which effectively overcomes the deficiency of the ground stub where the induced current along the Y direction is totally lacked. At the same time, the introduction of the thin horizontal stub inevitably increases the coupling energy between the antenna element and decoupling structure. To visualize the decoupling effect of the T-shaped ground branch, the surface electric field distribution of antenna 3 at resonant frequency is shown in Figure 9. Comparing Figure 8 with Figure 9 meticulously, it can be seen clearly that the coupling field existing in antenna 2 is immensely decreased, and a weak field area begins to appear between two antenna elements, which demonstrates that the above theoretical analysis and the modification linked to decoupling structure are reasonable. Comparing the transmission coefficients between the antenna with the I-shaped ground branch and antenna with the T-shaped ground branch, a tremendous reduction in transmission coefficient is achieved after modifying the structure of the parasitic element, which is in accordance with the surface field distribution analyzed above. The minimum transmission coefficient is close to −30 dB in antenna with the T-shaped ground branch; with reference to antenna 1, approximately 20 dB isolation improvement is realized after employing the T-shaped ground branch, which indicates that the decoupling effect of the T-shaped ground branch is superior to the I-shaped ground branch. However, the distinguished decoupling effect is at the sacrifice of impedance matching. As shown in Figure 3a, the reflection coefficient of the antenna is increased dramatically after modifying the structure of the parasitic element, which is due to the introduction of the T-shaped ground branch deteriorating the radiation environment of the antenna element.

#### 2.5.4. The Decoupling Effect of the Composite Parasitic Element

From the process of the I-shaped ground branch evolving into the T-shaped ground branch, it can be observed that the decoupling effect of the parasitic element is strongly associated with the intensity and distribution area related to the corresponding induced current, which can be used as a crucial guideline to further adjust the structure of the parasitic element. On the basis of the above discussion, there is no doubt that the induced current located on the T-shaped ground branch is mainly concentrated on the limited area of the bottom surface of the substrate, which is due to the planar physical structure of the T-shaped ground branch. Despite the fact that the field generated by the induced current located on the T-shaped ground branch can effectively neutralize the coupling field distributed on the bottom surface, there remains a problem that the coupling field inside the substrate and distributed on the top surface of the substrate can also constitute a comparatively weak coupling path. Hence, in order to further improve the isolation characteristic of the MIMO antenna, it is crucial to eliminate the residual coupling field inside the substrate and distributed on the top surface of the substrate. In reality, it is common knowledge that the conductive metal structures can perturb, constrain, or counteract electromagnetic waves. Therefore, they can potentially affect the field distribution leading to the reduction in the mutual coupling between antenna elements. In [38], the metal vias were integrated into the dielectric resonator antenna (DRA) to constrain the coupling field diffusing among two DRA elements. Inspired by this, a row of metal vias with constant period are integrated into the substrate of the antenna to further counteract the coupling field inside the substrate. As for the coupling field distributed on the top surface of the substrate, it can be effectively suppressed by adding an additional parasitic strip line printed on the center of the top surface of the substrate. Naturally, a new type of decoupling structure named the isolated branch is formed by connecting the parasitic strip and the ground stub of the T-shaped ground branch with the periodic metallic vias. Since the T-shaped ground branch and isolated branch share a common I-shaped ground branch, the combination of the T-shaped ground branch and the isolated branch can be considered as a composite parasitic element, which has a special three-dimensional physical structure.

**Figure 10:**
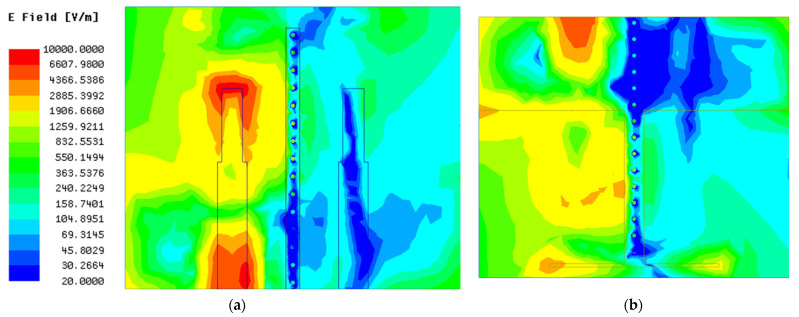
The distribution of surface electric field of antenna 4: (**a**) top surface; (**b**) bottom surface.

**Figure 11:**
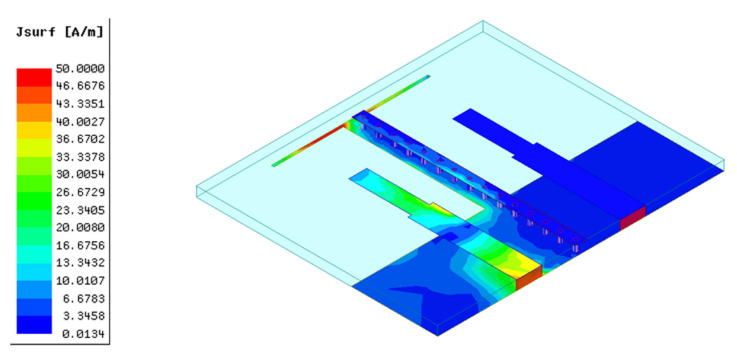
The current distribution of antenna 4.

To visualize the decoupling effect of this composite parasitic element, the surface electric field and current distribution of antenna with the composite parasitic element are shown in Figure 10 and Figure 11, respectively. Comparing the surface electric field of antennas with and without the isolated branch meticulously, it can be seen clearly that the distribution area of weak field between the two monopole elements is further enlarged after adjusting the structure of the parasitic element ingeniously. In addition, loading the isolated branch also promotes a decrease in the magnitude of the weak electric field area generated by the T-shaped ground branch. As shown in Figure 11, the induced currents distributed on the surface of T-shaped ground branch and parasitic strip line flow inside the metallic vias, corresponding to the blue dots in Figure 10. Otherwise, it can be observed that currents are scarcely distributed on the outer surface of the metallic vias, which demonstrates that the coupling current inside the substrate and induced current distributed on the outer surface of the metallic vias cancel each other out. From the association between the field and current, it can be easily inferred that the electric field scarcely exists around the metallic vias. Consequently, the coupling field inside the substrate is eliminated, resulting in a new weak electric filed area inside the substrate. Combining the weak electric fields on the top (generated by the parasitic strip line) and bottom (generated by the T-shaped ground branch) surfaces of the substrate, a unique three-dimensional weak electric field area is realized, which can block the coupling path between two monopole elements thoroughly in physical level. Hence, as presented in Figure 11, almost no coupling currents are distributed on element 2 when element 1 is excited.

To evaluate the decoupling effect of the composite parasitic element constituted by the T-shaped ground branch and the isolated branch, the transmission coefficients of the antenna at each phase are compared for quantitative analysis. As shown in Figure 3b, the highest isolation level of antenna with the composite parasitic element reached 42.9 dB. By contrast, the highest isolation level of antenna with T-shaped ground branch is close to 30 dB. In terms of the highest isolation level, an approximately 13 dB improvement was attained after loading the isolated branch, which immediately validates that the adjustment strategy associated with the decoupling structure mentioned above is feasible and reasonable. Conspicuously, the decoupling effect of the composite parasitic element is superior to the T-shaped ground branch, which is due to the fact that the three-dimensional weak electric field area realized by the composite parasitic element can eliminate the coupling source thoroughly compared to the two-dimensional weak electric field area generated by the T-shaped ground branch only. With reference to the antenna without any decoupling structure, the highest isolation improvement is approximately 32.7 dB after loading the composite parasitic element, and the average isolation improvement in total working frequency band is nearly 16 dB. It is noteworthy to mention that the isolated branch has little impact on the reflection coefficient of the MIMO antenna, which can be ascribed to the reason that the major physical structure of the isolated branch is integrated into the substrate. Comparing the reflection coefficients between antennas with and without the isolated branch, the reflection coefficient is changed from −22.6 dB to −20.3 dB after adding the isolated branch, which is a comparatively small variation in contrast to the design in previous two steps.

## 3. Parametric Analysis and Discussion

On the basis of the aforementioned analysis, there is no doubt that both the T-shaped ground branch and the isolated branch can immensely enhance the isolation characteristic of the proposed MIMO antenna. To further investigate the working mechanism of the decoupling structure and optimize antenna performance, several structural parameters of the parasitic elements that have significant influences on the antenna performance (reflection coefficient S11 and transmission coefficient S21) are implemented, including the diameter and periodic distance of the metallic vias and the length and width of the thin horizontal stub of the T-shaped ground branch. It is noteworthy to mention that only one structural parameter is varied each time and the rest are fixed.

### 3.1. Effect of the Metallic via Diameter d_2_ and Periodic Distance p

**Figure 12:**
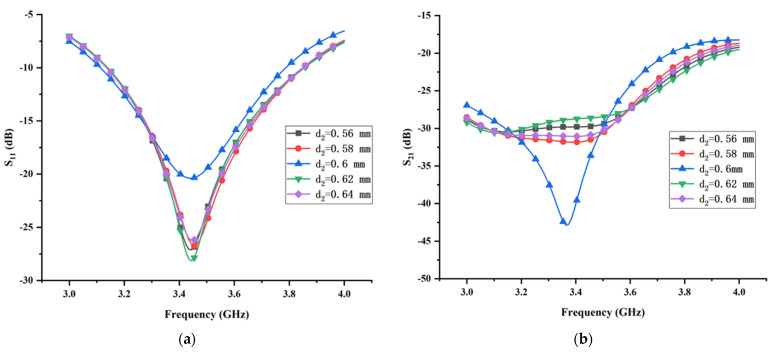
The effects of the diameters *d*_2_ of the metallic vias on (**a**) reflection coefficient and (**b**) transmission coefficient.

**Figure 13:**
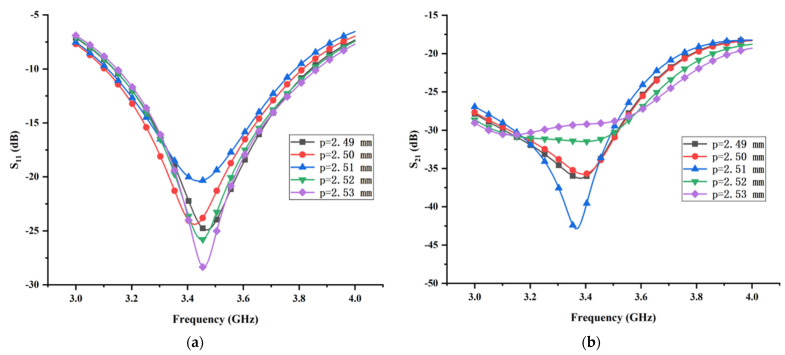
The effects of the periodic distance *p* of the metallic vias on (**a**) reflection coefficient and (**b**) transmission coefficient.

It can be expected that diameter *d*_2_ and periodic distance *p* may immensely affect the transmission coefficient of the proposed MIMO antenna, which can be explained in theoretical and physical aspects. In the theoretical aspect, as stated above, the combination of the T-shaped ground branch and isolated branch can be considered as a composite parasitic element. Hence, the variation of the diameter *d*_2_ and periodic distance *p* immediately affect the value of the coupling energy between the monopole element and the composite parasitic element. In the physical aspect, the induced field distribution around the metallic vias is mainly determined by the diameter *d*_2_ and the periodic distance *p*. Moreover, the diameter *d*_2_ and periodic distance *p* of the metallic vias can also affect the surface electric field distribution on the parasitic strip line and the T-shaped ground branch. In summary, the generation of the three-dimensional weak electric field area relies substantially on the diameter *d*_2_ and periodic distance *p*. Figure 12 presents the effect of the diameter *d*_2_ on the reflection coefficient and transmission coefficient with all other structural parameters fixed. It reveals that, as the diameter *d*_2_ increases gradually from 0.56 mm to 0.64 mm with constant step 0.02 mm, both the reflection coefficient and the transmission coefficient change dramatically. When *d*_2_ = 0.6 mm, the proposed MIMO antenna can obtain the optimal isolation, but the corresponding impedance matching is worse. Obviously, it is a tradeoff between the isolation and impedance matching. For the purpose of attaining the optimal isolation, the diameter *d*_2_ is set to 0.6 mm at the sacrifice of the impedance matching. The reflection coefficient and transmission coefficient versus the different periodic distance *p* are shown in Figure 13. It can be seen clearly that the reflection coefficient is significantly sensitive to the variation of the periodic distance *p*. When *p* = 2.51 mm, the corresponding impedance matching is the worst. By contrast, the optimal isolation can be obtained only when periodic distance *p* = 2.51 mm. Considering the best performance of isolation, the periodic distance *p* is chosen as 2.51 mm.

### 3.2. Effect of the Length L_6_ and Width W_6_ of the Thin Horizontal Stub

**Figure 14:**
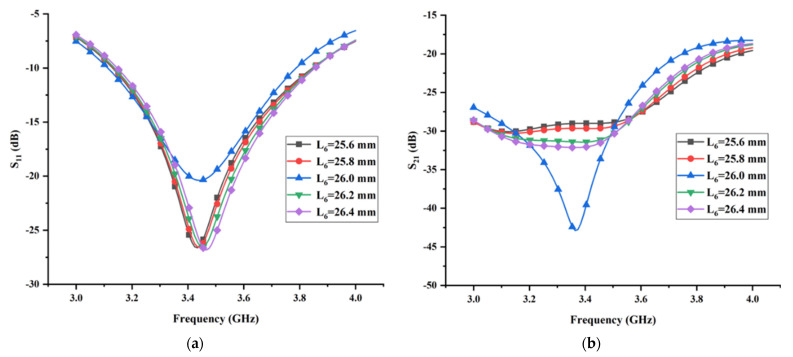
The effects of the length *L*_6_ of the thin horizontal stub on (**a**) reflection coefficient and (**b**) transmission coefficient.

**Figure 15:**
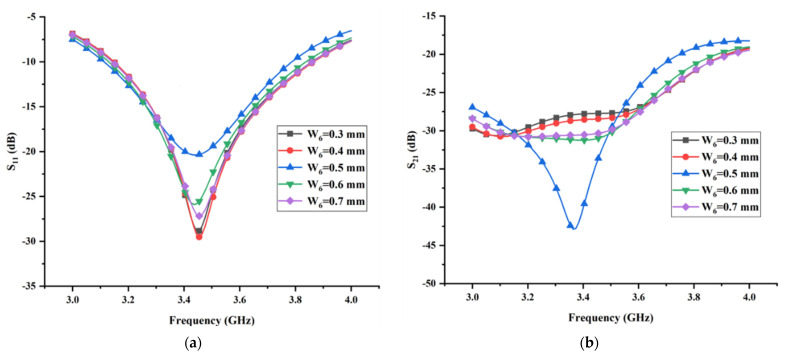
The effects of the width *W*_6_ of the thin horizontal stub on (**a**) reflection coefficient and (**b**) transmission coefficient.

As mentioned in the previous section, the isolation characteristic of the proposed MIMO antenna is improved drastically when the I-shaped ground branch is transformed into the T-shaped ground branch. Hence, the isolation of the proposed antenna is closely related to the structural parameters of the thin horizontal stub. The partial electromagnetic energy transmitting from the antenna radiating structure to the T-shaped ground branch can be equal to parallel resistant; hence, the variation of the structural parameters of the thin horizontal stub inevitably affects the impedance matching of the proposed antenna. Figure 14 and Figure 15 show the simulated reflection coefficient and transmission coefficient with different values of $L_{6}$ and $W_{6}$, respectively. As expected, both the reflection coefficient and the transmission coefficient are sensitive to the variation of the structural parameters of the thin horizontal stub. Among the five values of $L_{6}$ considered in Figure 14, $L_{6}$ = 26 mm is the only dimension resulting in the minimum transmission coefficient. Despite the fact that the case with $L_{6}$ = 26 mm can lead to the maximum reflection coefficient, considering that the priority of the isolation characteristic is higher the impedance matching in this design, the final dimension of $L_{6}$ is set as 26 mm. Similarly, from the performance exhibited in Figure 15 and in view of the minimum transmission coefficient in the whole operating band, the optimal value for $W_{6}$ was found to be 0.5 mm. The analysis procedure of the remaining structural parameters is similar to above, which is not be presented in this section for brevity.

## 4. Experimental Results

**Figure 16:**
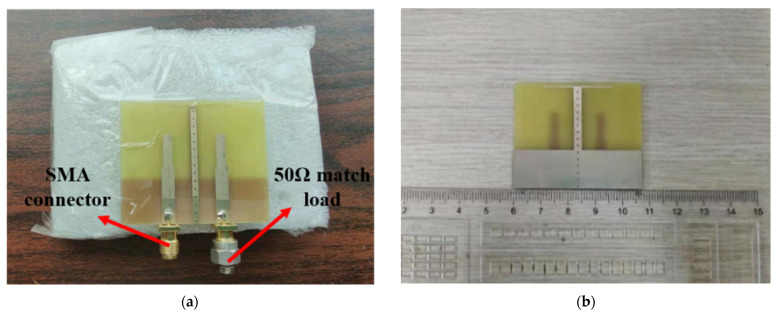
Photograph of the antenna prototype: (**a**) top view; (**b**) bottom view.

**Figure 17:**
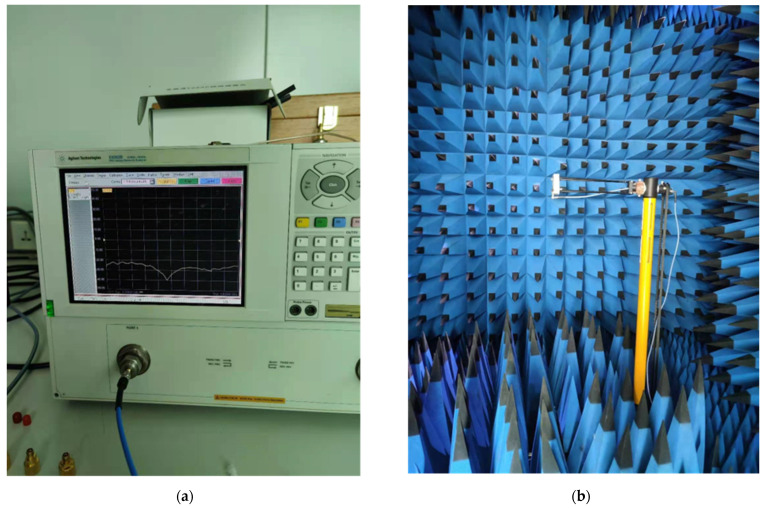
Measurement equipment: (**a**) vector network analyzer for scattering parameters; (**b**) anechoic chamber for radiation performance.

For validating the simulated results, the optimized design of the proposed MIMO antenna was fabricated using the standard printed circuit board technique with a size of 47.5 mm × 40 mm × 1.6 mm and a copper thickness of 0.035 mm. The photograph of the prototype of the proposed antenna is shown in Figure 16. It can be observed clearly from Figure 16 that the antenna is excited through two SMA connectors, whereby the inner conductors are linked with the feed line, and the outer conductors are linked to the ground layer. During the measurement, in addition to measuring the transmission coefficient, when one port was excited, the remaining port was terminated by a 50 Ω matched load. The measured environment of the proposed antenna in terms of the scattering parameters and radiation performance are shown in Figure 17a,b, respectively. As can be seen clearly in Figure 17, the scattering parameters were measured using an Agilent E8362B two-port vector network analyzer, while the radiation performance including gain and radiation pattern was measured in a far-field anechoic chamber using a standard horn antenna as a reference. To obtain an accurate measurement of radiation, the antenna was packaged with a hard foam board and fixed on the rotating platform in the anechoic chamber. Moreover, the antenna was kept away from absorber and metal material as best as possible.

### 4.1. Scattering Parameters

**Figure 18:**
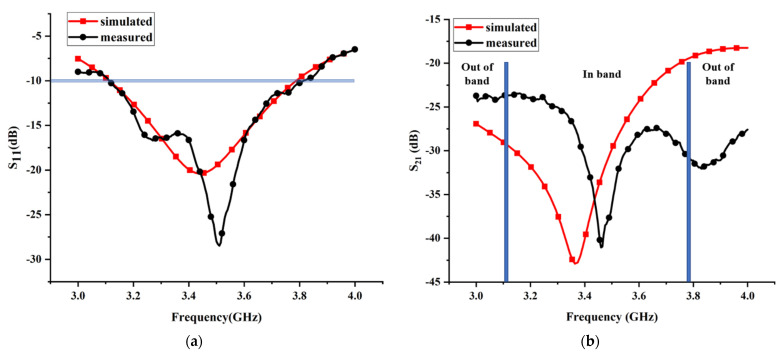
Measured and simulated S-parameters for the proposed MIMO antenna: (**a**) reflection coefficient; (**b**) transmission coefficient.

A comparison between the simulated and measured reflection coefficients (S_11_) is presented in Figure 18a. It can be observed from Figure 18a that the measured impedance bandwidth for S_11_ below −10 dB is around 20.2% (3.11–3.81 GHz) and the simulated impedance bandwidth for S_11_ below −10 dB is around 19.4% (3.11–3.78 GHz), testifying that the proposed antenna can cover the majority of frequency bands allocated for the sub-6 GHz spectrum of 5G communications. Obviously, the measured result is consistent with the simulated one in terms of the fractional bandwidth. However, the measured antenna resonant frequency is slightly shifted toward the higher frequency band (only about 0.06 GHz) compared to the simulated result, which might be attributed to minor fabrication and assembly inaccuracy. In Figure 18b, the simulated and measured transmission coefficients (S_21_) are exhibited. As can be seen clearly from Figure 18b, the measured minimum transmission coefficient is 41 dB, achieved at 3.46 GHz, whereas the simulated minimum transmission coefficient is 42.9 dB, achieved at 3.37 GHz. Compared to the simulated result, the measured minimum transmission coefficient is moved to the higher-frequency band, which can be ascribed to the fabrication error and measurement deficiency. Additionally, the measured minimum transmission coefficient is larger than the simulated one, which can be attributed to the reason that the weak mutual coupling between two cables of the measurement system is not considered in the simulation process. Nevertheless, the measured isolation is better than −23 dB across the entire operating band, which demonstrates that the proposed decoupling method is feasible. Overall, the measured transmission coefficient is imperfect but acceptable.

### 4.2. Radiation Performance

**Figure 19:**
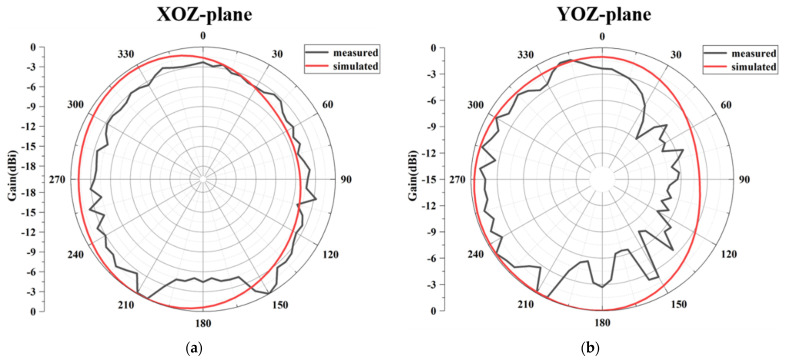
The simulated and measured normalized radiation patterns at 3.45 GHz: (**a**) XOZ plane; (**b**) YOZ plane.

**Figure 20:**
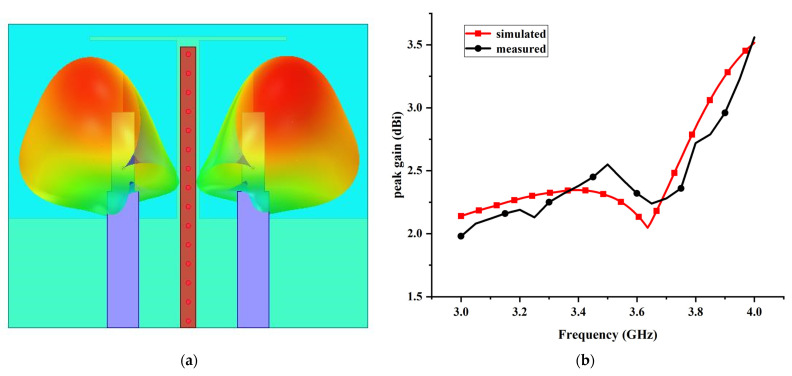
(**a**) The simulated 3D radiation pattern at 3.45 GHz; (**b**) the simulated and measured realized peak gain.

Figure 19 shows the simulated and measured normalized radiation patterns for the proposed MIMO antenna at 3.45 GHz, inclusive of the XOZ and YOZ planes. Figure 20a shows the simulated 3D radiation pattern of the antenna. A typical monopole-like omnidirectional radiation pattern is obtained in both XOZ and YOZ planes. This shows that the measured results are roughly consistent with the simulated, and the discrepancies are reasonable due to the fabrication error and the radiation from the test cable. As can be seen clearly, both simulated and measured radiation toward the +Y direction is slightly less than the −Y direction, especially in the measured result, indicating that the radiation around the decoupling structure is comparatively weak. This experimental phenomenon can be explained as follows: the radiation current in the three-dimensional weak field area generated by the composite parasitic element is extremely weak, leading to low radiation around the decoupling structure. Consequently, a low space correlation between the antenna elements is realized, which demonstrates that the proposed decoupling principle is reasonable.

Figure 20b illustrates the measured and simulated peak gains of the proposed MIMO antenna at each frequency. It can be observed apparently that the measured peak gain of the majority frequency points is slightly lower than the simulated values, which is mainly due to the loss of SMA connector and metallic material. By contrast, there is a small proportion of frequency points where the corresponding measured peak gain is larger than the simulated values, attributed to the fabrication and measurement error. However, the overall tendency of the measured peak gain curve is in accordance with the simulated result.

**Figure 21:**
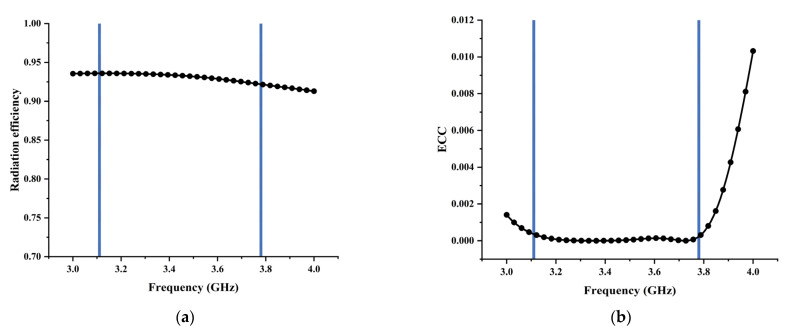
(**a**) The simulated radiation efficiency; (**b**) the calculated ECC.

Figure 21a shows the simulated radiation efficiency of the proposed antenna. The efficiency is obtained when element 1 is excited and element 2 is matched. It can be seen that the radiation efficiency in the working frequency band is above 0.9.

### 4.3. Envelope Correlation Coefficient (ECC)

The envelope correlation coefficient (ECC) illustrates how much MIMO antennas are independent in their individual performance, e.g., radiation patterns and polarization. It can be obtained from S-parameters or radiation patterns. In this paper, ECC was calculated from S-parameters as follows:

(2) $$\rho_{12}=\frac{|S_{11}^{*}S_{12}+S_{21}^{*}S_{22}{|}^{2}}{\left(1-{\left|S_{11}\right|}^{2}-{\left|S_{21}\right|}^{2}\right)\left(1-{\left|S_{22}\right|}^{2}-{\left|S_{12}\right|}^{2}\right)},$$

where $S_{ii}$ is the reflection coefficient, while $S_{ij}$ is the transmission coefficient. $S_{11}^{*}$ and $S_{21}^{*}$ are the complex conjugates of $S_{11}$ and $S_{21}$, respectively. Figure 21b shows the ECC of the proposed ^2^MIMO antenna. It can be seen that the ECC value is very low (less than 0.001) within the operating frequency. This indicates the proposed MIMO antenna system provides good diversity performance.

### 4.4. Diversity Gain (DG)

**Figure 22:**
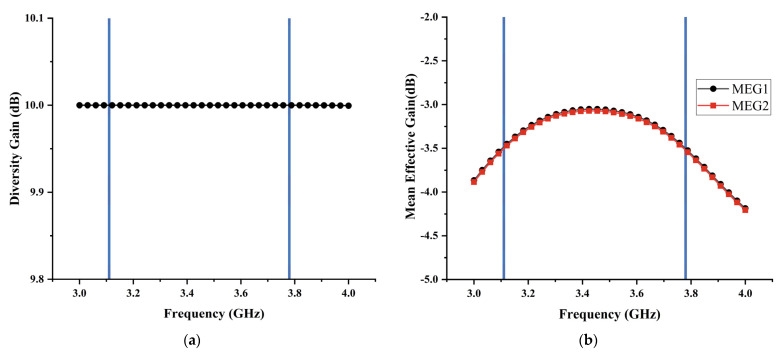
(**a**) The calculated DG; (**b**) The calculated MEG.

Diversity gain (DG) indicates the effect of the diversity scheme on the radiated power. Figure 22a shows the diversity gain of the proposed MIMO antenna which is calculated from the value of S-parameters using Equation (3) [43]. There is a flat line in the ideal value for 10 dB for the proposed MIMO antenna, which demonstrates the good diversity performance of the antenna.

(3) $$DG=10\sqrt{1-{\left|\rho_{\mathrm{ij}}\right|}^{2}},$$

where $\rho_{\mathrm{ij}}$ is the ECC calculated from S-parameters.

### 4.5. Mean Effective Gain (MEG)

The mean effective gain (MEG) is defined as the mean received power in the fading environment, which is another significant statistic to quantify diversity performance in MIMO systems. The acceptable practical value of MEG should be −3≤MEGi(dB)<−12. Figure 22b shows the MEG of the proposed MIMO antenna, which is less than −3 dB. Equation (4) is used to calculate the MEG values [44].

(4) $$MEGi=0.5\mu_{irad}=0.5\left(1-\sum_{j=1}^{K}\left|S_{ij}\right|\right),$$

where *K* is the number of antenna elements, i represents the antenna under observation, and $\mu_{irad}$ is the radiation efficiency.

## 5. Comparison with Previous Design

To better illuminate the superior features of the proposed high-isolation MIMO monopole antenna enabled by the parasitic element, a comprehensive comparison with previously reported designs in terms of some crucial technical indicators is summarized in Table 2 including electrical dimension, profile, edge separation, 20 dB isolation bandwidth, highest isolation level, and design complexity. In Table 2, edge separation is the spacing between the nearest edges of two antenna elements, and $\lambda_{0}$ is the wavelength of the central frequency. To ensure as fair a comparison as possible, the antenna types of the references listed in Table 2 are limited to the monopole antenna, which works in the low-frequency band. To make the comparison convincing, various decoupling methods are involved according to a common decoupling concept, including the neutralization line, decoupling network, electromagnetic bandgap, and parasitic element. As shown in Table 2, the advantageous features of the proposed MIMO antenna can be refined as follows:

(1) A comparative small dimension is achieved in our design, in which the corresponding electrical size is 0.55 λ_0_ × 0.46 λ_0_.
(2) A fabulous 20 dB isolation bandwidth is realized in this work, which reaches 19.4% with moderate edge separation.
(3) The highest isolation level reaches 42.9 dB in this work, which is far superior to other designs accompanied by low design complexity.

In [31,32], the high-isolation monopole array was realized by applying parasitic element. Both parasitic elements designed in [31,32] were planar structures, where the mutual coupling suppressing effect was inferior to the composite parasitic element designed in this work. Additionally, the design in [31] suffered from a large dimension and narrow 20 dB isolation bandwidth. The design in [32] had less isolation improvement in the operating frequency. In [29], the proposed wideband MIMO antenna had excellent isolation performance in the working frequency. However, despite obtaining a good 20 dB isolation bandwidth, the dimension of the antenna was relatively large. The design in [28] exhibited a low profile and small planar dimension, but it had deficiencies in that the 20 dB isolation bandwidth was excessively narrow and the isolation of the design was only 20 dB. In [26,27], the neutralization line was employed to realize the high isolation, exhibiting a distinguished 20 dB isolation bandwidth accompanied by relatively high design complexity. Although the 20 dB isolation bandwidth of the design in [26] was slightly wider than our design, its separation distance was twice as large and the corresponding highest isolation level was inferior.

To sum up, our proposed high-isolation MIMO monopole antenna shows advantages in terms of antenna dimension, design complexity, 20 dB isolation bandwidth, and highest isolation level. It is believed that the proposed decoupling method can be a potential candidate for future monopole array decoupling.

## 6. Conclusions

In this paper, a novel and effective decoupling method was proposed to eliminate the strong mutual coupling between two monopole antenna elements by inserting a composite parasitic element consisting of a T-shaped ground branch and the isolated branch. Specifically, unlike many previous parasitic elements, the designed parasitic element has a unique three-dimensional physical structure, which can eliminate the coupling field both inside and on the surface of the substrate. Consequently, a peculiar three-dimensional weak electric field area is generated, which can immensely suppress the mutual coupling between the antenna elements. The evolution and design principle of the composite parasitic element were illustrated meticulously step by step. In addition, the working mechanism of three types of parasitic elements involved in antenna design procedures was explained at both the physical and the theoretical level. To optimize the antenna performance, several structural parameters of the parasitic elements were implemented to attain the optimal dimension. Subsequently, to verify the feasibility of the proposed decoupling method, the antenna prototype was fabricated and measured. The measured results showed great agreement with the simulated results, indicating that the proposed decoupling method is feasible and reasonable. Lastly, a comprehensive comparison between this work and previous similar studies was implemented to illuminate the superior features of the proposed antenna. It was found that the proposed antenna has outstanding advantages including compact structure, fabulous highest isolation level, excellent 20 dB isolation bandwidth, and low design complexity.

## Figures and Tables

**Table 1:** Dimensions of the proposed MIMO antenna.

Parameters	$L_{1}$	$L_{2}$	$L_{3}$	$L_{4}$	$L_{5}$	$L_{6}$
Value (mm)	10.4	18	40	37	24	26
**Parameters**	$W_{1}$	$W_{2}$	$W_{3}$	$W_{4}$	$W_{5}$	$W_{6}$
Value (mm)	4.2	3	47.5	2	3	0.5
**Parameters**	$d_{1}$	$d_{2}$	P	h	$L_{7}$	
Value (mm)	13	0.6	2.51	1.6	14.36	

**Table 2:** Comparison of the proposed antenna with previous design.

Ref.	[26]	[27]	[28]	[29]	[31]	[32]	This Work
Antenna type	monopole	monopole	monopole	monopole	monopole	monopole	monopole
Method	NL	NL	DN	EBG	PE	PE	PE
Frequence (GHz)	3.3	2.3	2.4	6.9	2.6	3.5	3.45
Electrical dimension (λ0)	0.99 × 0.44	0.88 × 0.46	0.3 × 0.04	0.6 × 0.72	0.76 × 0.76	0.99 × 0.75	0.55 × 0.46
Profile (λ0)	0.009	0.006	0.003	0.018	0.25	0.005	0.018
Edge separation (λ0)	0.3	0.04	0.02	0.19	0.21	0.06	0.15
20 dB isolation BW (%)	26.7	11.1	12	109	1.5	5.7	19.4
Highest isolation level (dB)	36	35	20	60	28	30	42.9
Design complexity	Moderate	High	Low	Low	Moderate	Moderate	Low

## Data Availability

Not applicable.
